# Equine attachment site preferences and seasonality of common North American ticks: *Amblyomma americanum*, *Dermacentor albipictus*, and *Ixodes scapularis*

**DOI:** 10.1186/s13071-021-04927-8

**Published:** 2021-08-14

**Authors:** Kellee D. Sundstrom, Megan W. Lineberry, Amber N. Grant, Kathryn T. Duncan, Michelle M. Ientile, Susan E. Little

**Affiliations:** Department of Veterinary Pathobiology, College of Veterinary Medicine, Oklahoma State University, Stillwater, OK USA

**Keywords:** *Amblyomma*, Attachment site, *Dermacentor*, Equine, *Ixodes*, Tick

## Abstract

**Background:**

Ticks are common on horses, but recent publications characterizing equine tick infestations in North America are lacking.

**Methods:**

To further understand attachment site preferences of common ticks of horses, and to document the seasonality of equine tick infestation in northeastern Oklahoma, horses from eight farms were evaluated twice a month over a 1-year period. Each horse was systematically inspected beginning at the head and moving caudally to the tail. Attachment sites of ticks were recorded and all ticks collected were identified to species and stage.

**Results:**

Horses (26 males and 62 females) enrolled in the study ranged in age from 1 to 23 years (mean = 12, 95% CI 11–13). A total of 2731 ticks were collected; 84.1% (74/88) of the horses were infested (median = 3 ticks) at one or more examinations. Five tick species were identified, including *Amblyomma americanum* (78.2%; 2136/2731), *Ixodes scapularis* (18.2%; 497/2731), *Dermacentor albipictus* brown variant (2.6%; 71/2731), *Dermacentor variabilis* (0.7%; 20/2731), and *Amblyomma maculatum* (0.3%; 7/231). Most ticks were adults (83.6%; 2282/2731), but immature *A*. *americanum* (436/2136; 20.4%), *D*. *albipictus* (12/71; 16.9%), and *A*. *maculatum* (*n* = 1) were occasionally recovered. *Amblyomma americanum* were most often attached to the inguinal area, and *I*. *scapularis* and *D*. *albipictus* were most commonly found on the chest and axillary region (*P* < 0.0001). Ticks were found on horses in every month of the year. The largest number of ticks (638/2731; 23.4%) were collected in May (*P* < 0.0001). *Amblyomma americanum*, primarily immature, was the only tick recovered in September, *I*. *scapularis* and *D*. *albipictus* predominated October through February, and both *A*. *americanum* and *I*. *scapularis* were common in March. In the warmer months, April through August, *A*. *americanum* was the most common tick, followed by *D*. *variabilis* and *A*. *maculatum*.

**Conclusions:**

This research confirms that ticks common on horses in North America have attachment site preferences and that ticks infest horses in Oklahoma throughout the year, including during the winter. Additional research is warranted to fully understand the risk these infestations pose to equine health.

**Graphical abstract:**

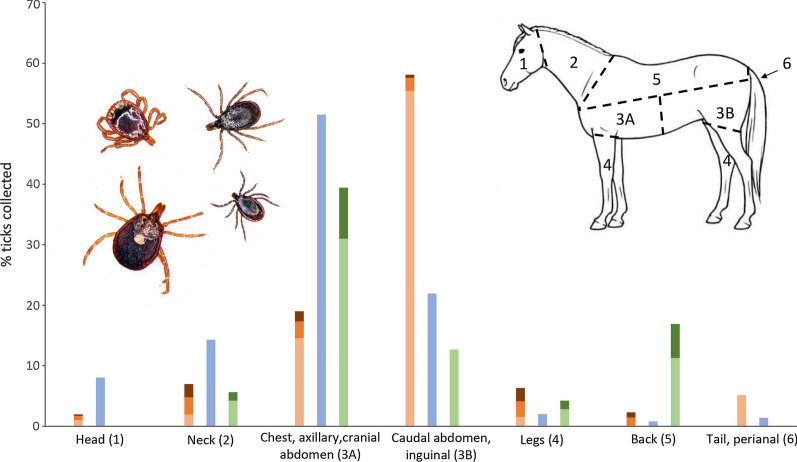

## Background

Ticks commonly infest horses in North America, causing localized inflammation and dermal trauma, and sometimes resulting in transmission of pathogens or systemic reactions [1–5]. Ixodid ticks most frequently identified from horses in the USA include *Ixodes scapularis*, which transmits *Borrelia burgdorferi* and *Anaplasma phagocytophilum*, both of which have been shown to cause equine disease; *Amblyomma americanum*, which can induce localized reactions; and *Dermacentor variabilis*, a species associated with equine tick paralysis and experimentally competent for transmission of *Theileria equi* [1, 3, 6–10]. Although considered of greater concern for cattle, horses in some areas of the USA can serve as hosts to *A*. *maculatum*, resulting in equine gotch ear, as well as the newly established *Haemaphysalis longicornis* and occasionally re-introduced *Rhipicephalus* (*Boophilus*) spp. ticks [6, 11–13]. Novel and recognized equine piroplasmosis agents have also been reported in the region [4, 14]. Despite the importance of ticks to equine health, recent comprehensive tick surveys of North American horses are sparse.

Different species of ticks display attachment site preferences on different hosts. For example, on dogs and cats, *I*. *scapularis* most commonly attaches to the head, ears, and neck, whereas *A*. *americanum* is more often found ventrally [15]. On white-tailed deer, over 85% of *I*. *scapularis* are attached to the ears, head, neck, and cranial thorax, while *A*. *americanum* are primarily found on the head and ears as well as ventrally on the abdomen, inguinal region, and perianal region; 75% of adult *A*. *americanum* are found attached to the left side of deer [1, 16]. On horses, most (84%) *I*. *scapularis* females are found attached to the chest, the axillary and inguinal regions, and below the chin, and adult *D*. *variabilis* prefer the tail and mane [1, 7]. In Israel, *Hyalomma* spp. and *Rhipicephalus turanicus* are most commonly attached to the inguinal region of horses, while *Haemaphysalis parva* is most often found on the neck and chest [17]. Equine attachment site preferences have not been reported for *A*. *americanum* or *D*. *albipictus*.

The seasonal activity of species and stages of ticks varies, leading to fluctuations in equine infestation risk throughout the year. An earlier survey conducted from May through July on Oklahoma horses described finding adult *A*. *americanum* earlier in the summer and adults of both *D*. *variabilis* and *A*. *maculatum* in later summer months [3]. *Ixodes scapularis* was collected from horses examined in Maryland from mid-October through November [1]. Tick collections from feral horses at Assateague Island National Seashore in April of 1987 and 1988 revealed mixed infestations with *A*. *americanum*, *D*. *variabilis*, and *I*. *scapularis*, although intensity of infestation was not reported and only the head and neck were examined [18]. Both *A*. *americanum* and *D*. *variabilis* were identified on horses in Kentucky examined May through August of 2008 [19]. Some ticks known to infest horses in North America, such as *I*. *scapularis* and *D*. *albipictus*, are active in the fall and winter, when few surveys are reported [1, 20]. To determine attachment site preferences of common equine ticks in North America, and to confirm the diversity and seasonal activity of ticks infesting horses in central Oklahoma, we examined horses on eight premises twice a month over the course of 12 months and collected and identified their ticks.

## Methods

Premises with horses were selected for participation based on the presence of a number of resident horses on-site; expected exposure to habitat with ticks was not considered. Horses were only enrolled if they resided on the premises, did not travel out of their county of residence, and were > 1 year of age at time of enrollment. All protocols were approved by the Institutional Animal Care and Use Committee at Oklahoma State University and permission to examine horses obtained through approved owner consent forms. Acaricide use was not specifically restricted or encouraged during the study, and owner acaricide practices were not recorded. Horses were examined for a full calendar year (12 months) from September 2019 through March 2021; horses continued to be enrolled as the study progressed, and tick exams were performed twice a month at each location for a full calendar year.

A total of 88 horses (26 males, 62 females) from eight different ranches in three counties in Oklahoma (Payne, Pawnee, Logan) were enrolled in the study, with ages ranging from 1 to 23 years (mean = 12, 95% CI 11–13). Several horses were sold or relocated in the course of the 1-year study; 54 horses were examined for a full 12 months, and 34 horses were examined 1–20 times for a total of 1661 equine tick examinations.

Horses were systematically examined beginning at the head and moving caudally to the tail, with an examiner positioned on either side of the horse. Once the dorsum and perianal regions were thoroughly scanned, the exam continued distally along the chest and forelegs, caudally along the axillary, ventral, and inguinal area, and ended moving along the distal hind legs [3]. Since horses were not sedated, exams did not include looking in ear canals. All stages of ticks were collected; when ticks were found, attachment site was recorded on a biopsy chart, ticks were removed, placed in snap cap vials, and labeled by horse and attachment site. Ticks were stored in 70% ethanol and identified to species and stage using standard morphological keys [20–26]. When necessary, morphologic identification was confirmed by *16S* rRNA gene sequence as previously described [15]. For data analysis, attachment sites were divided into seven body regions, namely head (1); neck (2); chest, axillary region, and cranial abdomen (3A); caudal abdomen and inguinal region (3B); legs (4); dorsal back (5); and tail and perianal region (6). Descriptive statistics [mean, range, proportion, and 95% confidence intervals (CI)] were calculated with Microsoft Excel (Microsoft Office Professional Plus 2016). Fisher’s exact test or Chi-square tests with a significance of alpha = 0.05 were used to compare age class and sex of infested horses, seasonality of infestation, and tick attachment site preferences, including ventral and dorsal, left and right, and body region.

## Results

### Ticks collected from horses

A total of 2731 ticks were collected. Over the entirety of the study, 84.1% of the horses (74/88; 95% CI 74.8–91.0%) were infested with ticks at one or more examinations (median = 3 ticks), consisting of 45.2% (1233/2731; 95% CI 43.3–47%) female ticks; 38.4% (1049/2731; 95% CI 37–40%) male ticks; 10.2% (279/2731; 95% CI 9.1–11.4%) nymphs; and 6.2% (170/2731; 95% CI 5.3–7.1%) larvae. Sex and age class of horses did not significantly influence tick infestation (Fisher’s exact test: *P* = 0.5426 and *P* = 0.6331, respectively).

The tick species identified were *A. americanum* (78.2%; 2136/2731; 95% CI 76.7–79.8%), *I. scapularis* (18.2%; 497/2731; 95% CI 16.8–19.7%), *D. albipictus* (2.6%; 70/2731; 95% CI 2–3.2%), *D. variabilis* (0.7%; 20/2731; 95% CI 0.5–1.1%), and *A. maculatum* (0.3%; 7/2731; 95% CI 0.1–0.5%). A majority of ticks collected (83.6%; 2282/2731; 95% CI 82.2–85%) were adults, but nymphs of *A*. *americanum* (12.5%; 266/2136; 95% CI 11–13.9%), *D*. *albipictus* (16.9%; 12/71; 95% CI 9.1–27.7%), and *A*. *maculatum* (14.3%; 1/7; 95% CI 0.36–57.9%) were also identified. *Amblyomma americanum* (8%; 170/2136; 95% CI 6.9–9.2%) were the only larval ticks collected (Table 1).

**Table 1:** Ticks collected from horses in northeastern Oklahoma by species, stage, and month of collection

Species	Stage	Total	Jan	Feb	Mar	Apr	May	Jun	Jul	Aug	Sep	Oct	Nov	Dec
*Amblyomma americanum*	F	794	0	0	34	186	255	190	81	45	1	0	2	0
	M	906	0	2	31	289	304	198	56	24	0	2	0	0
	N	266	0	0	1	20	68	57	5	41	73	1	0	0
	L	170	0	0	0	0	0	3	0	154	9	2	2	0
*Dermacentor albipictus*	F	52	5	2	0	1	0	0	0	0	0	0	16	28
	M	7	2	0	0	0	0	0	0	0	0	0	1	4
	N	12	0	0	0	0	0	0	0	0	0	0	9	3
	L	0	0	0	0	0	0	0	0	0	0	0	0	0
*Ixodes scapularis*	F	371	14	4	21	9	3	0	0	0	0	69	226	25
	M	126	2	0	4	0	0	0	0	0	0	16	97	7
	N	0	0	0	0	0	0	0	0	0	0	0	0	0
	L	0	0	0	0	0	0	0	0	0	0	0	0	0
Other^a^	F	16	0	0	0	1	3	9	2	1	0	0	0	0
	M	10	0	0	0	0	5	2	2	1	0	0	0	0
	N	1	0	0	0	1	0	0	0	0	0	0	0	0
	L	0	0	0	0	0	0	0	0	0	0	0	0	0
Total		2731	23	8	91	507	638	459	146	266	83	90	353	67

*F* female, *M* male, *N* nymph, *L* larva

^a^Other ticks collected from horses included *A*. *maculatum* and *D*. *variabilis*

### Attachment site preferences

**Fig. 1:**
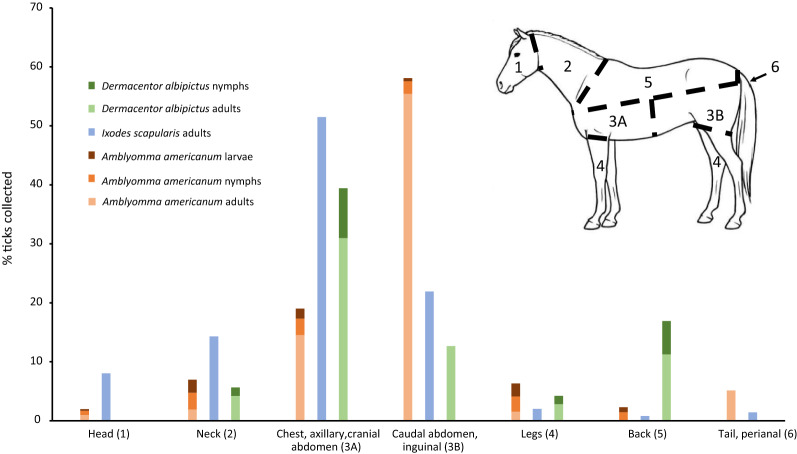
Attachment site preferences of *Amblyomma americanum*, *Ixodes scapularis*, and *Dermacentor albipictus* (brown variant) on horses

The attachment site differed between tick species collected. *Amblyomma americanum* adults were most often found in the inguinal area (Chi-square test: *χ*^2^ = 3372.28, *df* = 1, *P* < 0.0001), while nymphs were most commonly seen on the neck and chest (neck Chi-square test: *χ*^2^ = 16.174, *df* = 1, *P* < 0.0001; chest Chi-square test: *χ*^2^ = 14.80, *df* = 1, *P* < 0.0001) and larvae were most common on the neck and legs (neck Chi-square test: *χ*^2^ = 57.76, *df* = 1, *P* < 0.0001; legs Chi-square test: *χ*^2^ = 26.94, *df* = 1, *P* < 0.0001). *Ixodes scapularis* adults, the only stage of this species recovered from horses, and adults of *D*. *albipictus* were most commonly found on the chest and axillary region (*I. scapularis:* chest Chi-square test: *χ*^2^ = 561.48, *df* = 1, *P* < 0.0001; axillary Chi-square test: *χ*^2^ = 23.62, *df* = 1, *P* < 0.0001 and *D. albipictus*: chest Chi-square test: *χ*^2^ = 16.13, *df* = 1, *P* < 0.0001; axillary Chi-square test: *χ*^2^ = 15.53, *df* = 1, *P* < 0.0001); *D*. *albipictus* nymphs were also most common on the chest region (Chi-square test: *χ*^2^ = 12.480, *df* = 1, *P* = 0.0004) (Fig. 1). *Dermacentor albipictus* was more commonly identified on the left side (Chi-square test: *χ*^2^ = 5.085, *df* = 1, *P* = 0.024). Although ticks were more numerous on the left side, significant attachment side preferences (left versus right) were not evident for *A*. *americanum* (Chi-square test: *χ*^2^ = 3.303, *df* = 1, *P* = 0.069) or *I*. *scapularis* (Chi-square test: *χ*^2^ = 0.050, *df* = 1, *P* = 0.823). All three species with adequate numbers for evaluation [*A*. *americanum* (Chi-square test: *χ*^2^ = 1274.58, *df* = 1, *P* < 0.0001), *I*. *scapularis* (Chi-square test: *χ*^2^ = 384.24, *df* = 1, *P* < 0.000 1), and *D*. *albipictus* (Ch-square test: *χ*^2^ = 59.51, *df* = 1, *P* < 0.0001)] were significantly more likely to be attached ventrally.

### Seasonality of tick infestations

**Fig. 2:**
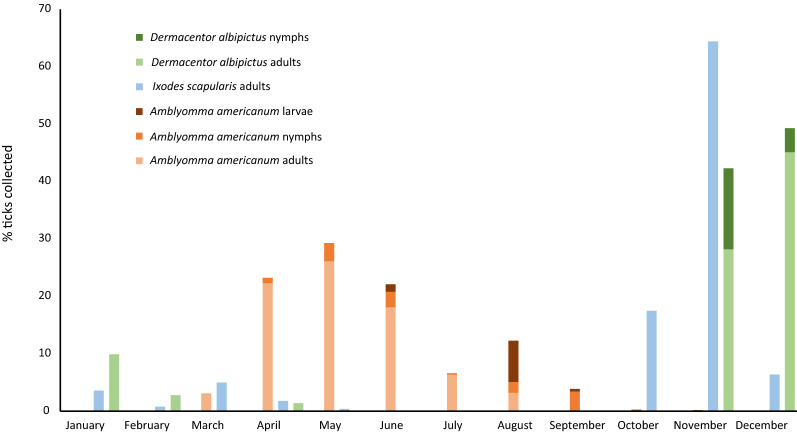
Seasonality of stages of *Amblyomma americanum*, *Ixodes scapularis*, and *Dermacentor albipictus* (brown variant) infesting horses, October 2019–March 2021

In fall, winter, and early spring (October 2019–March 2020; October 2020–March 2021), 633 ticks were collected, with 62.5% (55/88; 95% CI 51.5–72.6%) of the study population infested (median = 2 ticks) at one or more examinations. Tick species found during these cooler months included *I. scapularis* (76.8%; 486/633; 95% CI 73.3–80%), *D. albipictus* brown variant (11.1%; 70/633; 95% CI 8.7–13.8%), and *A. americanum* (12.2%; 77/633; 95% CI 9.7–15%) (Table 1, Fig. 2).

In the warmer months (April–September 2020), 2098 ticks were collected, with 72.7% (64/88; 95% CI 62.2–81.7%) of the study population infested (median = 3 ticks) at one or more examinations. Tick species found during these warmer months included *A*. *americanum* (98.2%; 2060/2098; 95% CI 97.6–98.8%), *D*. *variabilis* (1.0%; 20/2098; 95% CI 0.6–1.5%), and *A*. *maculatum* (0.3%; 7/2098 95% CI 0.1–0.7%) (Table 1, Fig. 2). Significantly more ticks (*P* < 0.0001; *X*^*2*^ = 785.9) were collected from horses in the warmer months (2098/2731; 76.8%; 95% CI 75.2–78.4%) than in the cooler months (633/2731; 23.2%; 95% CI 21.6–24.8%), but the prevalence of infestation did not differ between the two time periods (Chi-square test: *X*^*2*^ = 1.661, *df* = 1, *P* = 0.1973).

## Discussion

The present research confirms that, as reported for other hosts, attachment site of ticks on horses varies by tick species. This phenomenon is well recognized for *Otobius megnini*, a soft tick commonly found in the external ear canal of horses in the western USA, but less established for common ixodid species [27]. The finding in the present study that *I*. *scapularis* adults commonly attach to the chest of horses agrees with previously reported findings for horses in Maryland in the eastern USA [1]. In contrast, *A*. *americanum* was most frequently found in the inguinal region, similar to data from dogs and cats, confirming this species prefers to attach ventrally, and humans, where a majority of *A*. *americanum* attach below the waist [15, 28, 29]. A significant preference for the left side, as has been documented in white-tailed deer, was not evident in our *A*. *americanum* equine data [16]. However, the present study did document a left side bias for *D*. *albipictus* attachment, which has not, to our knowledge, been previously reported [30]. The basis for these attachment site preferences is not well understood but may involve differences in host-seeking strategies. *Amblyomma americanum* is more likely to aggressively move towards hosts along a carbon dioxide or pheromone gradient, emerging from the leaf litter to climb the legs and attach ventrally [27, 31]. In contrast, other tick species (e.g. *Dermacentor* spp., *Ixodes* spp.) employ a more passive questing strategy, climbing vegetation and waiting for a host to brush past [27, 32]. Together, these data and other studies indicate that ticks may be found on several regions of horses, supporting the need for complete external parasite examination when attempting to accurately characterize equine tick infestations [1, 17].

Similar to earlier reports from examining horses at certain times of the year, the most common tick species to infest horses in this region of North America, as in most of the eastern United States, are *A*. *americanum*, *I*. *scapularis*, and *Dermacentor* spp. [1, 3]. The predominance of *A*. *americanum* in the present study reflects the location of the study site in the southern USA where this tick is common [3, 33]. Similarly, the brown variant of *D*. *albipictus* (formerly referred to as *D*. *nigrolineatus*) and not the ornately patterned variety, is more commonly recovered from horses in this region although both variants have been reported [34]. *Ixodes scapularis* is also a frequent parasite of horses although only adult *I*. *scapularis* are routinely found on mammals in the southern USA [1, 35, 36]. Both *D*. *variabilis* and *A*. *maculatum* were relatively rare on horses in the present study, accounting for less than 1% of all ticks recovered, a finding likely due to the limited number of equine premises visited.

The data from the present study confirm that ticks infest horses year-round, including in the winter months (Table 1). To our knowledge, this is the first study confirming tick infestations on horses every month of the year in North America. As is seen in infestations in other domestic animals, the seasonality of equine infestations corresponds with established phenology for each tick species in the region, with *A*. *americanum* adults found in highest numbers on horses in the spring and summer, immature *A*. *americanum* most common in August and September, and both *D*. *albipictus* and *I*. *scapularis* predominating in the cooler fall and winter months [27, 28, 33]. Although a previous study suggested that both *D*. *variabilis* and *A*. *maculatum* are also commonly found on horses in the summer, the present study did not recover adequate numbers of either species to confirm this finding [3]. Seasonal differences in timing of peak activity between tick species leads to waxing and waning intensity of infestations, but tick populations do not entirely disappear, resulting in a year-round risk.

The findings from the present study have some limitations. Although occasionally identified in the region, *O. megnini* was not recovered from any horse, likely because this species is found in the external ear canal, and we did not sedate horses for thorough ear examination [27]. The low numbers of *A*. *maculatum* and *D*. *variabilis* recovered were surprising but may be due to the limited number of premises with horses enrolled (*n* = 8) or year-to-year fluctuations in tick populations related to precipitation or other factors [37]. Habitat may also have influenced the findings; both *A*. *americanum* and *I*. *scapularis* prefer wooded habitat with dense understory, whereas *D*. *variabilis* and *A*. *maculatum* are more commonly found in open areas, overgrown fields, or meadows [27]. In areas where Lyme disease is endemic or emerging, horses in pastures with oak trees are significantly more likely to be seropositive for *B*. *burgdorferi* [5]. Tick control practices were not recorded for the horses surveyed in the present study and likely varied between premises, but ticks were still commonly found (Table 1). Although attachment site preferences for the species considered are broadly applicable, additional research is warranted to fully appreciate the risk all tick species in North America pose to equine health. As with small animals, horses may benefit from year-round tick control. Unfortunately, available options for equine tick control are limited, require frequent re-application, and may have safety concerns, suggesting tick infestations will continue to be a challenge for horse owners.

## Data Availability

The data sets analyzed during the current study are available from the corresponding author on reasonable request.

## Abbreviations

*A*.: *Amblyomma*
*D.*: *Dermacentor*
*I*.: *Ixodes*
16S rRNA: 16S ribosomal RNA gene
CI: Confidence interval
df: Degrees of freedom
F: Female
M: Male
N: Nymph
L: Larva

## References

[1] Schmidtmann ET, Carroll JF, Watson DW (1998). Attachment-site patterns of adult blacklegged ticks (Acari: Ixodidae) on white-tailed deer and horses. J Med Entomol.

[2] Magnarelli LA, Ijdo JW, Van Andel AE, Wu C, Padula SJ, Fikrig E (2000). Serologic confirmation of *Ehrlichia equi* and *Borrelia burgdorferi* infections in horses from the northeastern United States. J Am Vet Med Assoc.

[3] Duell JR, Carmichael R, Herrin BH, Holbrook TC, Talley J, Little SE (2013). Prevalence and species of ticks on horses in central Oklahoma. J Med Entomol.

[4] Wise LN, Pelzel-McCluskey AM, Mealey RH, Knowles DP (2014). Equine piroplasmosis. Vet Clin North Am Equine Pract.

[5] Neely M, Arroyo LG, Jardine C, Moore A, Hazlett M, Clow K (2021). Seroprevalence and evaluation of risk factors associated with seropositivity for *Borrelia burgdorferi* in Ontario horses. Equine Vet J.

[6] Bishopp FC, Trembley HL (1945). Distribution and hosts of certain North American ticks. J Parasitol.

[7] Carroll JF, Schmidtmann ET (1986). American dog tick (Acari: Ixodidae), summer activity on equine premises enzootic for Potomac horse fever in south-central Maryland. J Econ Entomol.

[8] Stiller D, Goff WL, Johnson LW, Knowles DP (2002). *Dermacentor variabilis* and *Boophilus microplus* (Acari: Ixodidae): experimental vectors of *Babesia equi* to equids. J Med Entomol.

[9] Pusterla N, Chae JS, Kimsey RB, Berger Pusterla J, DeRock E, Dumler JS (2002). Transmission of *Anaplasma phagocytophila* (human granulocytic ehrlichiosis agent) in horses using experimentally infected ticks (*Ixodes scapularis*). J Vet Med B Infect Dis Vet Public Health.

[10] Trumpp KM, Parsley AL, Lewis MJ, Camp JW, Taylor SD (2019). Presumptive tick paralysis in two American miniature horses in the United States. J Vet Intern Med.

[11] Edwards KT (2011). Gotch ear: a poorly described, local, pathologic condition of livestock associated primarily with the Gulf Coast tick, *Amblyomma maculatum*. Vet Parasitol.

[12] Beard CB, Occi J, Bonilla DL, Egizi AM, Fonseca DM, Mertins JW (2018). Multistate infestation with the exotic disease-vector tick *Haemaphysalis longicornis*—United States, August 2017–September 2018. MMWR Morb Mortal Wkly Rep.

[13] United States Department of Agriculture. National *Haemaphysalis longicornis* (Asian longhorned tick) situation report, May 2021. https://www.aphis.usda.gov/animal_health/animal_diseases/tick/downloads/longhorned-tick-sitrep.pdf. Accessed 15 May 2021.

[14] Knowles DP, Kappmeyer LS, Haney D, Herndon DR, Fry LM, Munro JB (2018). Discovery of a novel species, *Theileria haneyi* n. sp., infective to equids, highlights exceptional genomic diversity within the genus *Theileria*: implications for apicomplexan parasite surveillance. Int J Parasitol.

[15] Saleh MN, Sundstrom KD, Duncan KT, Ientile MM, Jordy J, Ghosh P (2019). Show us your ticks: a survey of ticks infesting dogs and cats across the USA. Parasit Vectors.

[16] Bloemer SR, Zimmerman RH, Fairbanks K (1988). Abundance, attachment sites, and density estimators of lone star ticks (Acari: Ixodidae) infesting white-tailed deer. J Med Entomol.

[17] Tirosh-Levy S, Gottlieb Y, Apanaskevich DA, Mumcuoglu KY, Steinman A (2018). Species distribution and seasonal dynamics of equine tick infestation in two Mediterranean climate niches in Israel. Parasit Vectors.

[18] Oliver JH, Magnarelli LA, Hutcheson HJ, Anderson JF (1999). Ticks and antibodies to *Borrelia burgdorferi* from mammals at Cape Hatteras, NC and Assateague Island, MD and VA. J Med Entomol.

[19] Fritzen CM, Huang J, Westby K, Freye JD, Dunlap B, Yabsley MJ (2011). Infection prevalences of common tick-borne pathogens in adult lone star ticks (*Amblyomma americanum*) and American dog ticks (*Dermacentor variabilis*) in Kentucky. Am J Trop Med Hyg.

[20] Lindquist EE, Galloway TD, Artsob H, Lindsay LR, Drebot M, Wood H (2016). A handbook to the ticks of Canada (Ixodida: Ixodidae, Argasidae).

[21] Keirans JE, Litwak TR (1989). Pictorial key to the adults of hard ticks, family Ixodidae (Ixodida: Ixodoidea), east of the Mississippi River. J Med Entomol.

[22] Strickland RK, Gerrish RR, Hourrigan JL, Schubert GO. Ticks of veterinary importance, agriculture handbook No. 485. 1976. https://naldc.nal.usda.gov/download/CAT87208761/PDF. Accessed 15 May 2021.

[23] Cooley RA, Kohls GM (1944). The genus *Amblyomma* (Ixodidae) in the United States. J Parasitol.

[24] Cooley RA, Kohls GM (1945). The genus *Ixodes* in North America. US Natl Inst Health Bull.

[25] Keirans JE, Durden LA (1998). Illustrated key to nymphs of the tick genus *Amblyomma* (Acari: Ixodidae) found in the United States. J Med Entomol.

[26] Brinton EP, Beck DE, Allred DM. Identification of the adults, nymphs and larvae of ticks of the genus *Dermacentor* Koch (Ixodidae) in the western United States. Brigham Young Univ Sci Bull. 1965;5. https://scholarsarchive.byu.edu/byuscib/vol5/iss4/1. Accessed 15 May 2021.

[27] Saleh MN, Allen KE, Lineberry MW, Little SE, Reichard MV (2021). Ticks infesting dogs and cats in North America: biology, geographic distribution, and pathogen transmission. Vet Parasitol.

[28] Little SE, Barrett AW, Nagamori Y, Herrin BH, Normile D, Heaney K (2018). Ticks from cats in the United States: patterns of infestation and infection with pathogens. Vet Parasitol.

[29] Felz MW, Durden LA (1999). Attachment sites of four tick species (Acari: Ixodidae) parasitizing humans in Georgia and South Carolina. J Med Entomol.

[30] Samuel B (2004). White as a ghost: winter ticks and moose.

[31] Sonenshine DE (2018). Range expansion of tick disease vectors in North America: implications for spread of tick-borne disease. Int J Environ Res Public Health.

[32] Carroll JF, Allen PC, Hill DE, Pound JM, Miller JA, George JE (2002). Control of *Ixodes scapularis* and *Amblyomma americanum* through use of the ‘4-poster’ treatment device on deer in Maryland. Exp Appl Acarol.

[33] Paddock CD, Yabsley MJ (2007). Ecological havoc, the rise of white-tailed deer, and the emergence of *Amblyomma americanum*-associated zoonoses in the United States. Curr Top Microbiol Immunol.

[34] Patrick CD, Hair JA (1975). Ecological observations on *Dermacentor albipictus* (Packard) in eastern Oklahoma (Acarina: Ixodidae). J Med Entomol.

[35] Ghosh P, Saleh MN, Sundstrom KD, Ientile M, Little SE (2021). Ixodes spp. from dogs and cats in the United States: diversity, seasonality, and prevalence of *Borrelia burgdorferi* and *Anaplasma phagocytophilum*. Vector Borne Zoonotic Dis.

[36] Koch HG (1982). Seasonal incidence and attachment sites of ticks (Acari: Ixodidae) on domestic dogs in southeastern Oklahoma and northwestern Arkansas, USA. J Med Entomol.

[37] Paddock CD, Goddard J (2015). The evolving medical and veterinary importance of the Gulf Coast tick (Acari: Ixodidae). J Med Entomol.

